# The effects of a multi-ingredient dietary supplement on body composition, adipokines, blood lipids, and metabolic health in overweight and obese men and women: a randomized controlled trial

**DOI:** 10.1186/1550-2783-11-37

**Published:** 2014-07-26

**Authors:** Michael J Ormsbee, Shweta R Rawal, Daniel A Baur, Amber W Kinsey, Marcus L Elam, Maria T Spicer, Nicholas T Fischer, Takudzwa A Madzima, D David Thomas

**Affiliations:** 1 Department of Nutrition, Food and Exercise Sciences, Florida State University, Tallahassee, FL 32306, USA; 2 University of KwaZulu-Natal, Durban, South Africa; 3 Institute of Sports Science & Medicine, Florida State University, Tallahassee, FL, USA

**Keywords:** Green tea, Caffeine, Conjugated linoleic acid, Branched chain amino acids, Body composition, Obesity

## Abstract

**Background:**

The present study investigated the effects of a multi-ingredient dietary supplement (MIDS) containing caffeine, conjugated linoleic acid (CLA), green tea, and branched-chain amino acids (BCAA) taken for 8 weeks on body composition, blood lipid profile, glucose, insulin, adiponectin, leptin, and high-sensitivity C-reactive protein (hs-CRP) in overweight and obese men and women.

**Methods:**

Twenty-two participants completed the study (PL, n = 11; 7 women, 4 men; age, 34 ± 3.5 years; height, 169.2 ± 3.3 cm; body mass, 96.9 ± 6.8 kg; BMI, 34.1 ± 1.8 kg/m^2^; MIDS, n = 11; 9 women, 2 men; age, 36 ± 3.4 years; height, 173.2 ± 2.9 cm; body mass, 91.9 ± 5.6 kg; BMI, 30.0 ± 1.5 kg/m^2^). Participants were randomly assigned and stratified by body fat percentage to two groups: 1) a soybean oil placebo (PL) or 2) MIDS. Each group consumed two pills with breakfast and two pills with lunch. Body composition and android fat, waist and hip circumferences, blood pressure and heart rate were measured at baseline and after 8 weeks of supplementation.

**Results:**

There were no significant changes for any of the variables of body composition. Feelings of hunger were significantly higher in MIDS versus PL with no changes observed in satiety or desire to eat. Heart rate and blood pressure were unaltered in MIDS after 8 weeks of supplementation. Furthermore, lipid profile, food intake, mood state variables, fasting blood glucose, and endocrine markers did not significantly change regardless of group.

**Conclusion:**

MIDS intake does not appear to alter body composition or markers of cardiovascular health versus PL. Moreover, MIDS may actually increase feelings of hunger versus PL.

## Background

Obesity is a growing trend in the United States with current estimates suggesting prevalence as high as 35% among adults [1]. These high rates potentiate a severe health crisis as obesity increases the likelihood of developing chronic diseases which include hypertension, insulin resistance, type 2 diabetes mellitus (T2DM), arteriosclerosis, coronary heart disease, and metabolic syndrome [2]. Therefore, finding safe and effective methods for reducing body weight in obese individuals is essential.

Reducing body weight requires manipulation of the energy balance equation to produce energy deficits. This can be accomplished through diet and exercise, pharmacological interventions, or surgical means. However, each of these methods comes with disadvantages. For instance, many diet and exercise lifestyle interventions suffer from a lack of long-term (≥1 yr) adherence [3]. Furthermore, pharmacological and/or surgical means to reduce body weight are typically expensive and are sometimes accompanied by potentially unpleasant and/or dangerous side effects [4,5]. As such, consideration of alternative weight loss methods is warranted.

The consumption of natural ingredients and/or dietary supplements may provide a safe and effective means to induce weight loss and improve overall health. Indeed, recent evidence suggests that consumption of certain multi-ingredient dietary supplements may improve body composition and mood state [6,7]. The supplements utilized in these studies contained unique proprietary blends containing such ingredients as caffeine and green tea extract. These ingredients have been studied extensively and are now recognized as potential modulators of body weight, composition, adipokines, and metabolic health [8,9]. Beneficial effects of consuming green tea or caffeine, respectively, are likely a result of inhibited degradation of catecholamines (epinephrine and norepinephrine) or cyclic amino monophospates (cAMP) thereby enhancing thermogenesis and promoting lipolysis [10,11]. Of interest, the lipolytic effects reported with green tea and caffeine consumption may be the result of a synergistic effect as noted in a recent meta-analysis [12].

Supplementation with conjugated-linoleic acid (CLA) and branched-chain amino acids (BCAA) may also provide weight loss benefits [13,14]. These CLA-derived effects may be a result of enhanced β-oxidation via stimulation of enzymes responsible for transport of lipids into the mitochondria (i.e. carnitine palmitoyl transferase [CPT1]) [15], or through inhibition of adipocyte differentiation [16]. BCAA may improve body composition by enhancing protein synthesis, which maintains or increases lean mass [17]. While the efficacy of these ingredients has been extensively studied individually, there is a lack of research on the impact of combining these ingredients on body weight, composition and other markers of health. Furthermore, combining CLA and BCAA with green tea extracts and caffeine has also yet to be studied.

Therefore, the purpose of the present study was to examine the effects of a multi-ingredient dietary supplement (MIDS) containing a proprietary blend of green tea extracts, caffeine, CLA, and BCAA on body composition, blood lipid profile, glucose, insulin, adiponectin, leptin, and high-sensitivity C-reactive protein (hs-CRP) concentrations in overweight and obese men and women.

## Methods

### Participants

Thirty-four inactive (<2 times/week of planned physical activity for no more than 60 minutes per session) overweight or obese (BMI of 25 to 47 kg/m^2^) but otherwise healthy men and women, ages 18–50 years old were recruited from Tallahassee, Florida and surrounding areas. Prior to participation, each participant provided a written consent and completed a brief questionnaire regarding his or her medical and exercise training history. Participants were excluded if they were physically active (>2 times/week of planned physical activity for > 60 minutes), if they had uncontrolled hypertension (BP >140/90 mmHg), if they had high low-density lipoprotein cholesterol (LDL >160 mg/dL) or if they took cholesterol medication. In addition, those diagnosed with cardiovascular disease, stroke, diabetes, thyroid or kidney dysfunction, and smokers (>5 cigarettes per week) were excluded. Those who consumed any dietary supplements intended to alter body composition and body weight were excluded. In addition, those who had any allergies to soy, wheat, and grain products that would cause a health problem were excluded. This study was approved by Florida State University Institutional Review Board.

### Study design

This was a randomized, double-blinded, placebo-controlled study with two treatment groups. The participants were stratified based on body fat percentage and assigned to a placebo group (PL; soybean oil) or an isocaloric multi-ingredient dietary supplement group (MIDS; Suarez Corporation Industries, Canton, Ohio; 10 calories; 1 g fat, 99 mg caffeine, 1510 mg of a proprietary blend of: green tea, CLA, and BCAA per 2-pill serving [2 servings = 4 pills/day]). The green tea was standardized for 45% epigallocatechin gallate and 90% polyphenols. Both groups ingested 2 identical pills with both breakfast and lunch 7 days per week for 8 weeks.

All laboratory procedures were conducted following an 8 – 10 h overnight fast and a 24 h abstinence from caffeine, alcohol intake and any intense physical activity. Additionally, participants abstained from consuming their assigned treatment on testing days. Every visit was completed between 7 am and 10 am.

### Anthropometrics

Height and body mass were measured using a wall-mounted stadiometer and a digital scale (SECA, Birmingham, UK) every 2 weeks under identical conditions (shorts, t-shirt) for each subject. Waist and hip circumferences were obtained at baseline and after 8 weeks using a standard tape measure (with strain gauge) at the maximum area around the waist line and around the largest component of the gluteus maximus. All circumference measurements were conducted by the same researcher.

Total and regional body composition was determined by dual energy X-ray absorptiometry (iDXA; GE Lunar, Madison, WI) with participants in the supine position. Total body adiposity was expressed as percent body fat (% BF). Abdominal adiposity was determined by creating regions of interest (ROI) for the abdomen (region 1) using the ROI option within the manual analysis menu of the iDXA software and included the area just below the last rib to just above the iliac crest, as described previously [18]. Abdominal adiposity was expressed as percent android fat (% android fat). A certified radiologist technician performed all iDXA analyses.

Waist circumferences were measured at the maximum circumference around the umbilical point. Although this is not the standard measurement method for waist circumference, the abdominal obesity of many of the participants prevented the use of standard waist measurement techniques (i.e. 1–2 cm below the last rib typically corresponding with the narrowest part of the waist). Measurements were taken by the same researcher to minimize errors.

### Heart rate and blood pressure

Resting heart rate and blood pressure were measured at 0, 2, 4, 6, and 8 weeks. The participants were instructed to rest in a seated position, with both feet flat on the floor for 5 minutes before measurements were taken. Resting blood pressures were obtained by the same investigator on the same arm using an appropriately sized cuff and a sphygmomanometer (American Diagnostic Corp., Hauppauge, NY). Resting heart rate was obtained from a radial pulse. Two readings were taken for blood pressures and resting heart rate with 1 min intervals between each reading. The mean of the two readings were used for statistical analysis.

### Questionnaires: dietary intake, physical activity, hunger, and mood-state

Two-day diet logs were completed at weeks 0, 4, and 8 to quantify calorie and macronutrient intake. The participants were asked to maintain their food intake throughout the duration of the study. The Food Processor software (version 10.9 ESHA Research Salem, OR, USA) was used to analyze the food logs.

Participants were asked to not change their physical activity during intervention period. Physical activity logs completed by each participant were collected at weeks 0, 4 and 8. Adverse events forms were collected and recorded at weeks 2, 4, 6 and 8 by research staff.

Mood state was accessed via the completion of a 65-question mood state questionnaire which assessed mood state at weeks 0, 4 and 8. This was a simple likert scale (0, very – 4, not at all) questionnaire. Hunger was assessed at the same time points using a simple visual analog scale (VAS) (1 to 100 mm). Participants were instructed to place a mark on the 100 mm line to indicate their levels of hunger, satiety, and desire to eat. A mark at 0 indicated a complete lack of hunger, satiety, or desire to eat and a mark at 100 indicated extreme hunger, satiety, or desire to eat for each VAS, respectively. For each of the three measures (hunger, satiety, and desire to eat), the degree in which each sensation was felt was quantified by measuring how far the mark was from the 0 mm mark. For this measurement, a standard millimeter ruler was used and all scores were computed by the same investigator. VAS scales were completed at baseline (week 0) and post-intervention (week 8) in a fasted state.

### Hormones, blood lipids and blood glucose

A venous blood sample (20 ml) was obtained from the antecubital vein at baseline and after 8 weeks of supplementation (post). Whole blood was used to measure total cholesterol (TC), high-density lipoprotein cholesterol (HDL-C), low density lipoprotein cholesterol (LDL-C), triglycerides (TRG), and glucose concentrations using the Cholestech LDX blood analysis system (Hayward, CA). Inter-assay coefficients of variation were 2.1%, 4.0%, 4.1%, 4.7%, and 2.3% for TC, HDL, LDL, TRG, and glucose, respectively. Remaining samples were centrifuged (IEC CL3R Multispeed Centrifuge, ThermoElectron Corporation, Needham Heights, MA) for 15 minutes at 3500 rpm at 4°C. Serum aliquots of 300 μL were transferred into microtubes and stored at −80°C for later analysis of insulin, leptin, adiponectin, and hs-CRP. All assays were performed in duplicate in a single assay using commercially available ELISA kits according to the manufacturer’s instructions (leptin and adiponectin: R&D Systems Inc., Minneapolis, MN, USA; hs-CRP and insulin: IBL International, Inc., Hamburg, Germany). The mean of duplicate samples were used for statistical analysis. Inter-assay coefficients of variation were 1.3%, 6.2%, and 5.2% for leptin, adiponectin, and hs-CRP, respectively. Inter-assay data were missing for insulin due to technical errors. Intra-assay coefficients of variation were 5.6%, 13.8%, 3.8%, and 8.1% for insulin, leptin, adiponectin, and hs-CRP respectively. Insulin resistance was assessed using the homeostatic model of assessment, as described previously [19].

### Compliance

Compliance was checked by asking participants to bring supplement containers every 4 weeks. Weekly calls or emails were sent to remind participants to take the supplement and maintain their food and physical activity logs for the duration of the study.

### Statistical analysis

An *a priori* power analysis was performed which revealed a need for a minimum of 6 participants per group to achieve a power of 0.80, α = 0.05, standard deviation = 1.1, difference = 6 (41). One way analysis of variance (ANOVA) was performed to examine possible group differences at baseline. Data were analyzed using a 2X5 repeated measures ANOVA ([PL × MIDS]) × ([week 0 × week 2 × week 4 × week 6 × week 8]). Data were analyzed using JMP PRO 9 software (Cary, NC). If significant main effects were identified by ANOVA, a Tukey post hoc comparison test was performed to locate differences.

## Results

### Participant demographics

A total of 160 individuals were pre-screened for participation in this study. Of the 68 individuals eligible for the study, 34 decided to participate. Out of these 34 participants, 5 participants withdrew from the study due to personal reasons. A total of 29 participants completed the study, however, data from 7 participants was excluded due to low compliance (<80% of total supplement intake). Therefore, 22 participants with 11 in each group were included in the statistical analysis. Aside from fat mass (p<0.05), there were no statistical differences between groups at baseline (Table 1).

**Table 1:** Participant demographics at week 0 (N = 22)

**Variables**	**PL [n = 11 (7 women, 4 men)]**	**MIDS [n = 11 (9 women, 2 men)]**	***p***
**Age (years)**	33. 8 ± 3.5	35.9 ± 3.4	0.67
**Height (cm)**	169.2 ± 3.3	173.2 ± 2.9	0.36
**BM (Kg)**	96.9 ± 6.8	91.9 ± 5.6	0.57
**% BF**	43.6 ± 2.5	40.5 ± 2.4	0.45

Data are mean ± SEM. PL, Placebo; MIDS, multi-ingredient dietary supplement; BM, body mass; BMI, body mass index; %BF, percent body fat; FM, fat mass; FFM, fat free mass; W/H ratio, wait to hip ratio.

### Body composition

**Figure 1:**
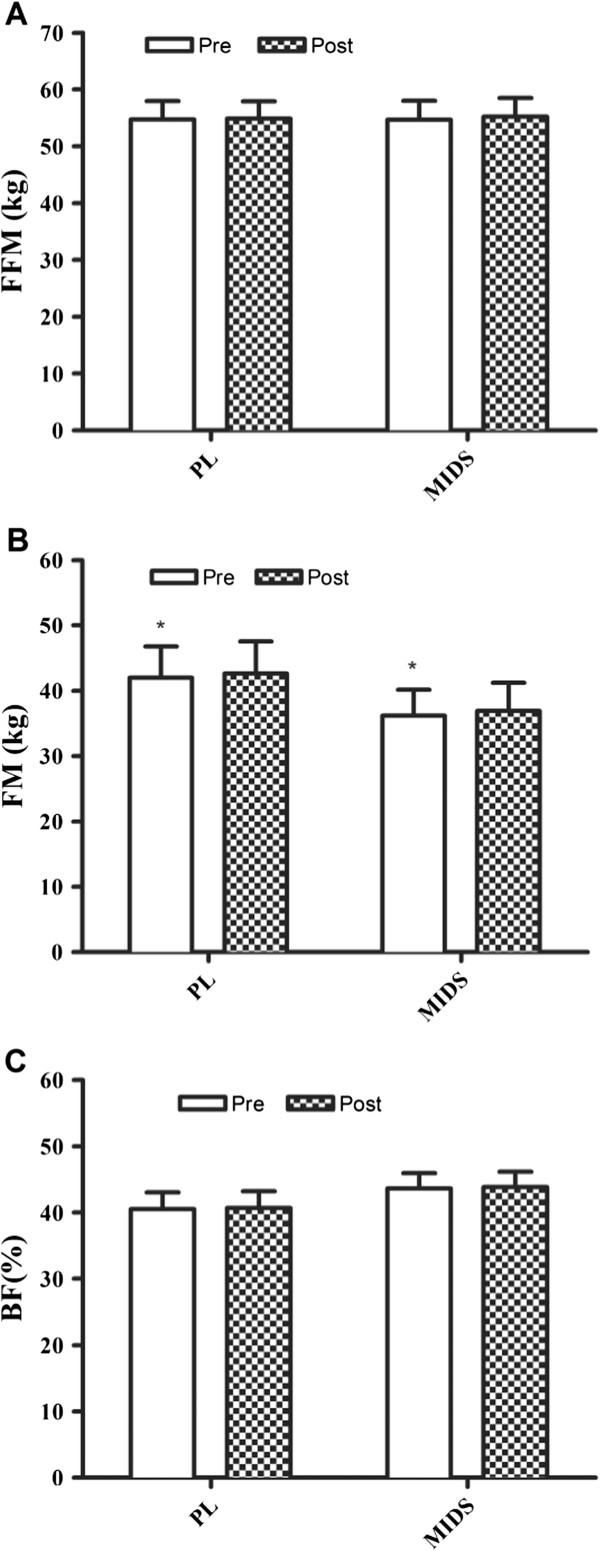
**Free-fat mass, fat mass, and percent body fat changes pre- to post-intervention. A**, free fat mass pre- and post-intervention. **B**, fat mass pre- and post-intervention. **C**, body fat percent pre- and post-intervention. FFM, free-fat mass; kg, kilogram; PL, placebo; MIDS, multi-ingredient dietary supplement; FM, fat mass; BF, body fat; %, percent. *denotes that groups were significantly (P < 0.05) different at week 0 (pre).

Body composition and anthropometric data is presented in Figure 1 and Table 2. There were no main time or group × time interactions observed for the following variables: body mass, body mass index, percent body fat, % android fat, percent gynoid fat, fat mass, fat free mass, waist and hip circumference, and waist to hip ratio. However, a significant time effect was observed for BMI (+1.2 and +1.0%) and FM (+1.7 and +1.9%) for PL and MIDS, respectively (Table 2). Additionally, post hoc analyses revealed no differences between genders.

**Table 2:** Body composition and anthropometrics at week 0 and week 8

**Variables**	**Group**	**n**	**Week 0**	**Week 8**	**∆**	**Time** ***p***	**Time × group** ***p***
**BM (kg)**	PL	11	96.9 ± 6.8	97.9 ± 7.1	+1.0	0.24	0.70
	MIDS	11	91.9 ± 5.6	92.4 ± 5.9	+0.5		
**BMI (kg/m**^**2**^**)**	PL	11	34.1 ± 1.8	34.5 ± 1.9	+0.4	0.02	0.62
	MIDS	11	30.0 ± 1.5	30.3 ± 1.5	+0.3		
**% BF**	PL	11	43.6 ± 2.5	43.8 ± 2.4	+0.2	0.38	0.91
	MIDS	11	40.5 ± 2.4	40.7 ± 2.6	+0.2		
**% Android fat**	PL	11	49.9 ± 3.2	49.2 ± 2.8	−0.7	0.85	0.07
	MIDS	11	44.8 ± 2.8	45.5 ± 3.1	+0.7		
**% Gynoid fat**	PL	11	46.5 ± 2.9	46.7 ± 2.7	+0.2	0.63	0.23
	MIDS	11	43.0 ± 2.6	42.6 ± 2.8	−0.5		
**FM (kg)**	PL	11	41.9 ± 4.8	42.6 ± 4.9	+0.7	0.04	0.84
	MIDS	11	36.2 ± 4.0	36.9 ± 4.3	+0.7		
**FFM (kg)**	PL	11	54.7 ± 3.2	54.9 ± 3.0	+0.2	0.24	0.50
	MIDS	11	54.7 ± 3.3	55.2 ± 3.3	+0.5		
**Waist (cm)**	PL	11	107.0 ± 4.3	105.2 ± 3.9	−1.8	0.32	0.69
	MIDS	11	99.9 ± 3.2	99.1 ± 3.3	−0.8		
**Hip (cm)**	PL	11	121.0 ± 4.9	120.6 ± 4.7	−0.5	0.59	0.90
	MIDS	11	114.0 ± 3.3	113.7 ± 3.6	−0.2		
**Waist to hip ratio**	PL	11	0.89 ± 0.02	0.88 ± 0.02	−0.01	0.48	0.73
	MIDS	11	0.88 ± 0.02	0.87 ± 0.03	−0.01		

Data are mean ± SEM; PL, Placebo; MIDS, multi-ingredient dietary supplement; BM, body mass; BMI, body mass index; % BF, percent body fat; % Android fat, percent Android fat; % Gynoid fat, percent gynoid fat; waist, waist circumference; hip, hip circumference.

∆ change from week 0 to week 8.

### Lipid profile, heart rate, and blood pressure

**Figure 2:**
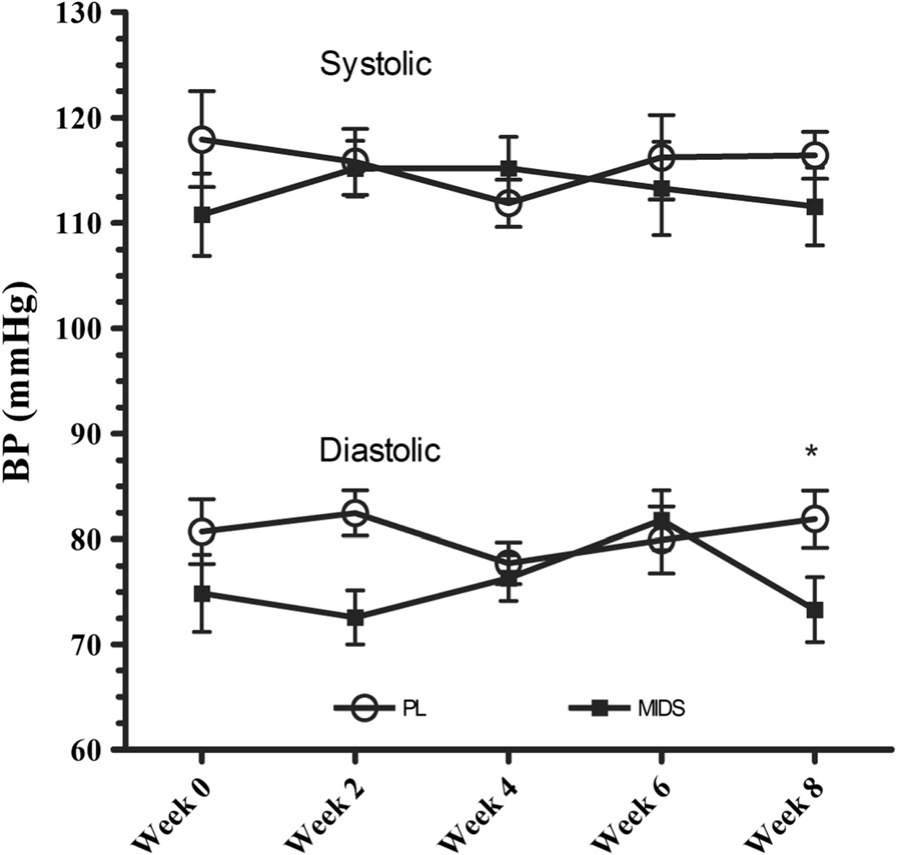
**Systolic and diastolic blood pressure over time.** BP, blood pressure; mmHg, miligrams of mercury; PL, placebo; MIDS, multi-ingredient dietary supplement. *denotes that PL at week 8 was significantly (p < 0.05) different than MIDS at weeks 0, 2, 4, and 8.

The lipid profile variables measured were total cholesterol, high density lipoprotein, low density lipoprotein and triglycerides. There was no significant main time or group × time interactions observed for any variables of the lipid profile or fasting blood glucose (Table 3). We observed a main time and group × time effect for heart rate. Specifically, heart rate increased in PL and was unchanged in MIDS (PL, ∆ + 4.0 bpm vs. MIDS, ∆ 0 bpm, p = 0.005). There was also a main time and group × time effect for diastolic blood pressure (PL, ∆ + 2 mmHg vs. MIDS, ∆ -1 mmHg, p = 0.04) with no changes in systolic blood pressure (Figure 2).

**Table 3:** Lipid profile at week 0 and week 8

**Variables**	**Group**	**n**	**Week 0**	**Week 8**	**∆**	**Time** ***p***	**Time × group** ***p***
**TC (mg/dl)**	PL	11	179.7 ± 7.9	183.8 ± 8.8	+4.0	0.39	0.91
	MIDS	11	178.9 ± 8.6	184.2 ± 14.2	+5.0		
**TRG (mg/dl)**	PL	10	119.5 ± 18.7	129.7 ± 23.4	+9.0	0.99	0.19
	MIDS	11	107.0 ± 19.3	96.9 ± 21.1	−10		
**HDL (mg/dl)**	PL	11	56.1 ± 5.1	50.6 ± 5.1	−5.0	0.39	0.27
	MIDS	11	52.4 ± 3.6	53.1 ± 3.9	+1.0		
**LDL (mg/dl)**	PL	10	102.1 ± 10.7	109.3 ± 7.4	+7.0	0.12	0.96
	MIDS	11	105.1 ± 8.6	111.8 ± 12.1	+7.0		
**LDL/HDL**	PL	10	2.2 ± 0.4	2.4 ± 0.3	+0.2	0.14	0.68
	MIDS	11	2.1 ± 0.3	2.3 ± 0.4	+0.1		

Data are mean ± SEM; PL, Placebo; MIDS, multi-ingredient dietary supplement; TC, total cholesterol; TRG, triglycerides; HDL, high density lipoprotein; LDL, low density lipoprotein; HR, heart rate; SBP, systolic blood pressure; DBP, diastolic blood pressure; n is different for TRG, LDL, LDL/HDL as a result of insufficient data.

∆ change from week 0 to week 8.

### Food intake

Dietary intake of total calories, carbohydrates, proteins, and fats was not different at baseline and did not change after 8 weeks of supplementation regardless of group. Data are presented in Table 4.

**Table 4:** Food intake at week 0 and week 8

**Variables**	**Group**	**n**	**Week 0**	**Week 8**	**∆**	**Time** ***p***	**Time × group** ***p***
**Calories (kcal)**	PL	10	2666.2 ± 283.1	2366.9 ± 343.1	−299.3	0.36	0.41
	MIDS	11	2846.0 ± 244.6	2478.5 ± 377.8	−367.5		
**Carbohydrate (g)**	PL	10	335.2 ± 37.1	271.35 ± 39.8	−63.85	0.27	0.63
	MIDS	11	333.5 ± 37.3	299.0 ± 55.1	−34.53		
**Carbohydrate (%)**	PL	10	50.9 ± 2.8	46.3 ± 3.3	−4.6	0.31	0.54
	MIDS	11	46.9 ± 3.2	47.9 ± 2.5	+1.0		
**Protein (g)**	PL	10	93.0 ± 10.6	97.2 ± 16.2	+4.2	0.27	0.60
	MIDS	11	112.3 ± 10.0	95.4 ± 12.1	−16.9		
**Protein (%)**	PL	10	14.8 ± 1.7	16.6 ± 1.4	+1.8	0.88	0.55
	MIDS	11	16.8 ± 2.1	16.3 ± 1.5	−0.5		
**Fat (g)**	PL	10	101.9 ± 14.7	91.9 ± 18.0	−10	0.55	0.06
	MIDS	11	119.9 ± 15.8	101.6 ± 16.6	−18.3		
**Fat (%)**	PL	10	33.1 ± 2.1	34.4 ± 3.2	+1.8	0.13	0.08
	MIDS	11	37.1 ± 2.5	36.6 ± 2.3	−0.5		

Data are mean ± SEM; PL, Placebo; MIDS, multi-ingredient dietary supplement.

∆ change from week 0 to week 8.

### Hunger scale

**Figure 3:**
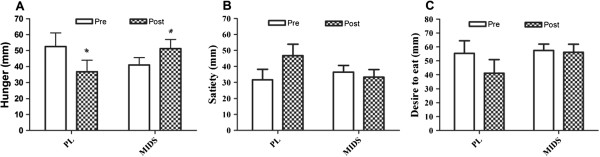
**Hunger, satiety, and desire to eat ratings pre- and post-intervention. A**, hunger ratings pre- and post-intervention. **B**, satiety ratings pre- and post-intervention. **C**, desire to eat ratings pre- and post-intervention. mm, milimeters; PL, placebo; MIDS, multi-ingredient dietary supplement. *denotes a group × time interaction (p < 0.05).

Ratings of hunger, satiety, and desire to eat are presented in Figure 3. No significant group × time interactions or time effects were observed for satiety or desire to eat. However, a significant group × time interaction was observed for hunger (PL, ∆ - 15.8 mm vs. MIDS, ∆ + 10.3 mm, p = 0.04).

### Mood state

No significant group × time interactions or time effects were observed for any of the mood state variables. However, a main time effect (p = 0.02) was observed for tension (PL, ∆ - 3.78 vs. MIDS, ∆ – 2.2) and confusion (PL, ∆ -3.2; vs. MIDS, ∆ -1.2).

### Hormones

Fasting concentrations of insulin, leptin, adiponectin, and hs-CRP are presented in Table 5. No significant time effects or group by time interactions were observed for any of the measured hormones. Additionally, insulin resistance was not different between groups over time and no significant changes were observed in either group after 8 weeks of treatment.

**Table 5:** Endocrine changes at week 0 and week 8

**Variables**	**Group**	**n**	**Pre**	**Post**	**∆**	**Time** ***p***	**Time × group** ***p***
**Insulin (μIU/mL)**	PL	11	18.8 ± 3.6	17.2 ± 1.5	−1.6	0.57	0.16
	MIDS	10	11.4 ± 1.2	15.1 ± 2.0	+3.7		
**Leptin (pg/mL)**	PL	11	52.2 ± 11.4	42.0 ± 8.1	−10.2	0.13	0.58
	MIDS	11	30.7 ± 7.6	25.9 ± 7.5	−4.8		
**Adiponectin (μg/mL)**	PL	11	5.9 ± 0.8	6.5 ± 1.2	+0.6	0.73	0.12
	MIDS	11	9.8 ± 1.7	8.9 ± 1.5	−0.9		
**hs – CRP (mg/L)**	PL	11	4.4 ± 1.1	4.6 ± 1.3	+0.2	0.89	0.37
	MIDS	10	2.8 ± 0.9	2.5 ± 0.6	−0.3		
**Glucose (mg/dl)**	PL	11	99.0 ± 3.3	98.2 ± 4.4	- 0.8	0.94	0.78
	MIDS	11	96.3 ± 4.3	96.9 ± 5.0	+0.6		
**HOMA-IR**	PL	11	4.66 ± 0.98	4.19 ± 0.42	−0.47	0.73	0.19
	MIDS	10	2.82 ± 0.44	3.61 ± 0.52	+0.81		
**A/L**	PL	11	0.36 ± 0.1	0.44 ± 0.1	+0.08	0.13	0.39
	MIDS	11	1.1 ± 0.2	1.3 ± 0.23	+0.2		

Data are mean ± SEM; PL, Placebo; MIDS, multi-ingredient dietary supplement; hs-CRP, high sensitivity C reactive protein; HOMA-IR, homeostatic model assessment insulin resistance; A, Adiponectin; L, Leptin; *n* is different for insulin and hs-CRP as data was missing due to technical errors.

∆ change from week 0 to week 8.

No significant group by time interactions or time effects were observed for adiponectin to leptin (A/L) ratio for either group (Table 5). However, A/L ratios were significantly higher in MIDS at baseline and post-intervention compared to PL.

### Reported side effects

In PL, there was a report of insomnia (n = 1) and bloating (n = 1). Side effects reported for MIDS were acne (n = 1), jitteriness (n = 1), nausea (n = 1), weight gain (n = 1), and loss of appetite (n = 1). No other side effects were reported in either group.

## Discussion

The primary findings of this study were that 8 weeks of MIDS supplementation in overweight/obese men and women did not reduce body mass or improve body composition versus PL. Furthermore, endocrine markers (insulin, leptin, adiponectin, and hs-CRP), blood lipids, satiety, food intake, and mood state variables were not significantly different or after 8 weeks regardless of group.

The results of the present study are in opposition to recent reports of enhanced body composition, mood state, and mental focus with the consumption of other multi-ingredient dietary supplements [6,7]. In these studies, supplements were consumed which contained green tea extracts and caffeine among other ingredients. As MIDS shared these ingredients in common with the supplements used in these studies, the lack of improvement in body composition was unexpected.

Indeed, research examining green tea and caffeine either alone or in combination have consistently reported these ingredients to reduce body mass and fat mass in overweight or obese individuals [8,20-22]. Moreover, the mechanisms for these effects are also well-researched. Caffeine stimulates the release of catecholamines and inhibits degradation of cAMP, which enhances sympathetic stimulation [11,23]. Similarly, green tea can also increase thermogenesis as it contains caffeine and numerous polyphenols (catechins, epicatechin, epigallocatechin, and their gallates). Epigallo Catechin Gallate (EGCG), the most abundant polyphenol in GT, prevents catecholamine degradation via inhibition of catechol-o-methlytransferase (COMT) enzyme activity [10]. This, similar to the effects of caffeine, can effectively prolong norepinephrine-induced sympathetic stimulation thereby enhancing thermogenesis [10].

The lack of significant change in body mass or composition can be explained by a number of factors. Interestingly the impact of caffeine may be attenuated by the development of tolerance. Robertson et al. [24] demonstrated that caffeine (250 mg/day) increased plasma catecholamines after 3 days of ingestion in healthy men and women. However, after 14 days of continuous supplementation the plasma catecholamine concentrations decreased and were comparable to the placebo [24]. The participants in our study were moderate consumers of caffeine (~137 mg/day as self-reported; range: 0 to ~618 mg/day), possibly resulting in attenuated sensitivity to caffeine supplementation during the trial period. The lack of change in heart rate and blood pressure in MIDS appears to confirm this. The ineffectiveness of caffeine in the current study may also be related to genetic factors. Indeed, Womack et al. [25] noted a genetic polymorphism which influences one’s responsiveness to caffeine in terms of exercise performance. Perhaps the majority of subjects in our study were ‘non-responders’ to caffeine although this is purely speculation. Additionally, a meta-analysis on the body mass and composition effects of caffeine and GT noted ethnicity to be a significant factor [26]. Indeed, caffeine and GT may have more pronounced effects on Asians versus Caucasians. This may be due to genetic differences in COMT activity [27]. As our study was composed of various ethnicities (e.g. Caucasian, African American, and Asian), this may have increased variability resulting in the observed non-significant effects. Finally, the composition of the multi-ingredient dietary supplements utilized in other studies [6,7] is significantly different from MIDS. Thus, the influence of other ingredients may explain the benefits reported in these studies. Another possibility is that the ingredients in these other supplements may synergistically enhance body composition. Alternatively, the potential benefits of the green tea and caffeine in MIDS may have been counteracted by some of its other ingredients, but the experimental design did not allow for examination of the weighted-effects of the various ingredients.

Our findings of a lack of change in body mass or composition is also somewhat unexpected based on reported benefits of CLA ingestion. CLA, a fatty acid abundant in seed oils, may aid in the regulation of lipid metabolism. Specifically, CLA has been reported to reduce uptake of lipids into adipocytes by inhibiting gene expression and activity of stearoyl-CoA desaturase and lipoprotein lipase [28,29]. Additionally, CLA seems to increase the activity of CPT1 thereby increasing fatty acid oxidation. These mechanisms may explain improvements in body composition that have been consistently reported with CLA supplementation in animals [28,30].

There is some evidence to suggest that CLA improves body composition in humans. Blankson et al. [31], studied overweight/obese participants that consumed various amounts of CLA (1.7, 3.4, 5.1, 6.8 g/day) following exercise for 12 wks. As a result of the intervention, participants who received the highest dose of CLA (6.8 g/day) increased lean mass to a greater degree than the other groups. In addition, decreases in fat mass were reported in the groups receiving 3.4 and 6.8 g/day CLA, (but not 5.1 g/day). Others have also reported enhanced lean mass regain following weight loss in overweight participants with 1.8 to 3.4 g/day of CLA [32] and reduced % BF in healthy, normal weight, individuals with 4.2 g/day of CLA [33]. With this in mind, our findings of no change in body mass or composition could be the result of sub-optimal doses of CLA. In the current study, the proprietary blend consumed in MIDS combined the dose of CLA with BCAA (2.52 g/day composed of CLA and BCAAs). Thus, even if the majority of the proprietary blend was composed of CLA, the dose would still likely fall at the low-end of reported effective dosages (1.8-6.8 g/day). Alternatively, it is also possible that the benefits of CLA are only realized when combined with exercise [31]. Participants in our study were inactive prior to and during the study, which may have influenced the effectiveness of CLA. Whatever the case may be, our findings are in line with numerous others reporting that CLA supplementation has no effect on body mass or composition [34-36]. Further study on the true effects of CLA on body mass and composition in humans in clearly warranted.

In the present study, body composition was not improved despite the BCAA content of MIDS. Increasing dietary protein helps to reduce weight, and preserve lean body mass in healthy adults [37], and some of the benefits may be attributable to BCAA [38]. These effects may be a function of the stimulatory effect that certain BCAA (e.g. leucine) have on muscle protein synthesis [17]. Positive effects from BCAA consumption on body composition are often reported when combined with exercise [39]. However, the influence of BCAA on body composition in inactive individuals is relatively unknown. Our findings suggest that consuming additional BCAA (<2.52 g/day) in combination with the other ingredients in MIDS has no effect on body composition. Alternatively, perhaps a higher dose is required to realize potential benefits. Indeed, many animal studies reporting BCAA-induced losses in fat mass utilize >5 g/day [40].

Maintaining low levels of LDL, TC, and TRG and high levels of HDL may help to prevent cardiovascular disease [41]. Our findings do not support MIDS as being cardio-protective as we observed no changes in blood lipids. Some studies have reported caffeine, green tea, and/or CLA to reduce LDL, TC, and TRG while maintaining or increasing HDL [22,31,42]. However, the doses used in these studies were typically higher than in the current study and/or were combined with exercise or dietary interventions. Perhaps more robust dietary and/or physical activity changes are required to see significant changes in blood lipids.

Endocrine biomarkers were similarly unaffected by MIDS or PL. This was not altogether unexpected as the ingredients in MIDS may have opposing effects on the hormones measured. For instance, caffeine and CLA are known to reduce insulin sensitivity [43,44]. However, caffeine and green tea have also been reported to increase adiponectin, a hormone produced in adipose that may prevent insulin resistance [45,46]. These opposing effects may explain why insulin and fasting glucose were unchanged with MIDS. Leptin, a hormone that may regulate energy intake, and hs-CRP, a proinflammatory cytokine implicated in atherogenesis, were similarly unchanged following MIDS supplementation. Although there is some evidence suggesting that CLA may reduce leptin levels [47], the effects of the other ingredients on leptin and hs-CRP are relatively unknown. The results of the current study suggest that MIDS consumption does not alter leptin, hs-CRP, or hormones related to insulin sensitivity.

Satiety was also unaffected by MIDS supplementation. When attempting to lose weight, increasing satiety may help to reduce energy intake. Previous studies have reported enhanced satiety with increased dietary protein intake [37]. Additionally, caffeine supplementation may reduce spontaneous energy intake [48]. Nevertheless, we observed no changed in satiety or food intake. As mentioned, our subjects were moderate caffeine consumers, which likely attenuated any caffeine-mediated effects on satiety. Moreover, participants in the current study did not increase dietary protein intake, but rather supplemented with a relatively small dose of BCAA. Perhaps BCAAs-alone may have minimal effects on satiety, or the dose was too small to elicit any changes in satiety. It is worth noting that participants consuming MIDS reported an increase in hunger. A study by Chiou et al. [49] reported that participants taking a purported dietary supplement, which was actually a non-caloric placebo, showed a preference for a buffet meal rather than an organic meal. Furthermore, subjects receiving the sham supplement also showed reduced desire to exercise [49]. In the current study, it is possible that participants that believed they were receiving a dietary supplement felt increased license for hedonic behavior possibly contributing to the increased hunger ratings. However, it should be noted that in the present study participants in either group did not increase food intake. Thus, further study on the true effects of MIDS on satiety and hunger are warranted.

In conclusion, consumption of MIDS for 8 wks in overweight and obese, but otherwise healthy, men and women resulted in no changes in body mass or composition, blood lipids, endocrine biomarkers, mood state, or satiety. In future studies, larger doses of MIDS should be administered, and the effects of MIDS in combination with diet and/or exercise should be investigated.

## Abbreviations

MIDS: Multi-ingredient dietary supplement; PL: Placebo; GT: Green tea; CLA: Conjugated linoleic acid; BCAA: Branched chain amino acids.

## Competing interests

The authors declare that they have no competing interests.

## Authors’ contributions

MJO designed and managed the study, secured funding, analyzed the data and drafted the manuscript. SRR carried out all practical aspects of the study and assisted with data analysis, and manuscript preparation. MTS assisted with study design and manuscript preparation. DAB, AWK, MEE, NF, TAM, and DT assisted data collection and analysis and manuscript preparation. All authors read and approved the final manuscript.
